# An orienting response is not enough: Bivalency not infrequency causes the bivalency effect

**DOI:** 10.2478/v10053-008-0142-9

**Published:** 2013-09-20

**Authors:** Alodie Rey-Mermet, Beat Meier

**Affiliations:** Institute of Psychology and Center for Cognition, Learning, and Memory, University of Bern, Switzerland

**Keywords:** bivalent stimuli, task switching, cognitive control, episodic context binding

## Abstract

When switching tasks, occasionally responding to bivalent stimuli (i.e., stimuli with relevant features for two different tasks) slows performance on subsequent univalent stimuli, even when they do not share relevant features with bivalent stimuli. This performance slowing is labelled the *bivalency effect*. Here, we investigated whether the bivalency effect results from an orienting response to the infrequent stimuli (i.e., the bivalent stimuli). To this end, we compared the impact of responding to infrequent univalent stimuli to the impact of responding to infrequent bivalent stimuli. For the latter, the results showed a performance slowing for all trials following bivalent stimuli. This indicates a long-lasting bivalency effect, replicating previous findings. For infrequent univalent stimuli, however, the results showed a smaller and shorter-lived performance slowing. These results demonstrate that the bivalency effect does not simply reflect an orienting response to infrequent stimuli. Rather it results from the conflict induced by bivalent stimuli, probably by episodic binding with the more demanding context created by them.

## INTRODUCTION

Imagine yourself driving home. You drive quickly but safely, and when you approach a red traffic light, you put one foot on the brake pedal and the other on the clutch. After years of practice, your behaviour is guided by unambiguous environmental cues. However, what would you do if both the red and the green traffic lights were lit at once? The purpose of this study was to shed light on the cognitive processes you engage in when encountering such ambiguous stimuli.

In a laboratory task, one way to investigate this type of situation is to use bivalent stimuli. Bivalent stimuli have relevant features for two different tasks and thus they induce a conflict about which task to perform (e.g., Jersild, 1927; Meiran, 2008). To resolve this conflict, an adjustment of cognitive control is required in order to select task-relevant features while suppressing distracting ones (Botvinick, Braver, Barch, Carter, & Cohen, 2001; Botvinick, Cohen, & Carter, 2004). Recent studies have shown that the occasional occurrence of bivalent stimuli triggers an adjustment of cognitive control for subsequent performance. That is, *occasionally* encountering bivalent stimuli slows performance on several subsequent univalent stimuli, even on those that share no relevant features with the bivalent stimuli (Meier, Rey-Mermet, Woodward, Mueri, & Gutbrod, 2013; Meier, Woodward, Rey-Mermet, & Graf, 2009; Rey-Mermet, Koenig, & Meier, 2013; Rey-Mermet & Meier, 2012a, 2012b; Woodward, Meier, Tipper, & Graf, 2003; Woodward, Metzak, Meier, & Holroyd, 2008).

In these studies, participants performed triplets of binary tasks on univalent stimuli, with bivalent stimuli occasionally occurring in one task (see Meier & Rey-Mermet, 2012a, for a review). For example, they were instructed to repeatedly perform a parity decision (odd vs. even) on black numerals, a colour decision (red vs. blue) on red or blue symbols, and a case decision (uppercase vs. lowercase) on black letters. For some case decisions, the letters were presented in red or blue print colour, thus turning them into bivalent stimuli. The results revealed a performance slowing for all tasks following bivalent stimuli, including those with stimuli that shared no relevant stimulus features with the bivalent stimuli (i.e., the parity decisions).

This performance slowing, coined the *bivalency effect*, has now been demonstrated with different types of tasks, different types of bivalent stimuli, across different modalities, and with overlapping as well as with non-overlapping response sets (Meier et al., 2009; Metzak, Meier, Graf, & Woodward, in press; Rey-Mermet & Meier, 2012a). Furthermore, it is not affected by a manipulation of the interval between task triplets (i.e., 1,000 ms, 2,000 ms, 3,000 ms, or 5,000 ms) and it persists across at least four subsequent purely univalent triplets, that is, for more than 20 s (Meier et al., 2009). The bivalency effect has also been associated with activation in the dorsal anterior cingulate cortex, a brain area recruited for the adjustment of cognitive control (Grundy et al., 2013; Woodward et al., 2008). In an event-related potential (ERP) study, it was associated with an ERP component reflecting interference in cognitive control (Rey-Mermet et al., 2013). Moreover, amnesic patients did not show the long-lasting slowing typical for the bivalency effect. They only showed a short-lived slowing on the task that immediately followed the bivalent stimulus (Meier et al., 2013).

Together, these findings indicate that the bivalency effect reflects a robust and long-lasting adjustment of cognitive control following the conflict induced by bivalent stimuli. Critically, the current cognitive control accounts cannot explain the bivalency effect, because these accounts focus primarily on processes initiated by the stimulus, response, or task features (see Allport & Wylie, 2000; Egner, 2007; Hommel, 2004; Rogers & Monsell, 1995; Waszak, Hommel, & Allport, 2003). According to these accounts, after encountering a bivalent stimulus, bivalent stimulus features would be activated on the univalent trials that share a feature with the bivalent stimulus. These bivalent stimulus features would be inhibited, because they are irrelevant for task execution (Allport, Styles, & Hsieh, 1994; Allport & Wylie, 1999, 2000; Wylie & Allport, 2000). Alternatively, they would require an additional task-decision process in order to select the relevant task (Braverman & Meiran, 2010; Fagot, 1994; Meiran, Kessler, & Adi-Japha, 2008; Rogers & Monsell, 1995). In both cases, performance would be slowed but only for the univalent trials sharing relevant features with the bivalent stimuli. However, the bivalency effect is also found on the univalent trials sharing no relevant features with the bivalent stimuli (e.g., Meier et al., 2009; Rey-Mermet & Meier, 2012a; Woodward et al., 2003). Therefore, the cognitive control accounts cannot fully explain the bivalency effect.

To account for the bivalency effect, we put forward the hypothesis that the bivalency effect is due to “episodic context binding” (Meier et al., 2009, 2013; Meier & Rey-Mermet, 2012a). As we found no bivalency effect for amnesic patients, we reasoned that most likely, this effect results from episodic binding (see Meier et al., 2013). However, as the bivalency effect occurs irrespective of stimulus, response, or task overlap (Rey-Mermet & Meier, 2012a; Woodward et al., 2003), this binding must go beyond stimulus, response, and task features, and thus extends to the particular context. Thus, extending the notion that a stimulus is bound to the task in which it occurs (Waszak et al., 2003), we have suggested that the stimulus and the task are bound to the context in which they occur (i.e., episodic context binding). In the particular paradigm used to assess the bivalency effect, from the perspective of the participant, the context consists of all the three decision tasks (rather than just one of them). Thus, responding to these tasks in a given order creates a specific context and, concurrently, binds the tasks and the univalent stimuli to this context. As the three tasks are presented repeatedly, this specific context is reactivated constantly. However, responding to a task with a bivalent stimulus makes the context more demanding. For subsequent decisions, the representation of the - now conflict-loaded - context is reactivated. This interferes with processing the tasks with purely univalent stimuli (Rey-Mermet et al., 2013), slowing down performance and resulting in the bivalency effect. Thus, according to the “episodic context binding” account, the bivalency effect reflects interference caused by the reactivation of the more demanding context created by bivalent stimuli.

An alternative explanation would be that bivalent stimuli capture attention simply because they occur infrequently (cf. Notebaert et al., 2009; Notebaert & Verguts, 2011; Nůńez Castellar, Kühn, Fias, & Notebaert, 2010). The resulting orienting response endures across a few subsequent trials, slowing down performance on univalent trials. Thus, the bivalency effect might represent an orienting response towards infrequent events. Evidence in favour of such an interpretation can be derived from a study of Notebaert et al. (2009). In one of their experiments, participants had to perform a four-colour choice reaction time task. Colour intensity was adjusted in order to reach predefined accuracy levels. In a 75%-accuracy condition, colour intensity was set such that 75% of the responses were correct, and consequently, correct responses were frequent events whereas errors were infrequent events. In contrast, in a 35%-accuracy condition, 35% of the responses were correct, and consequently, errors were frequent events whereas correct responses were infrequent events. The results showed a performance slowing after an infrequent event, irrespective of whether this event was an error or a correct response. In another experiment, Notebaert et al. presented tones after a response was made. Here, the tones were oddball in 25% of the trials. The results showed a performance slowing after the oddball tones. Thus, in both experiments, an after-effect was found after an infrequent event, suggesting that the low frequency of the event was critical (cf. Notebaert & Verguts, 2011; Nůńez Castellar et al., 2010; see also Barcelo, Escera, Corral, & Periáńez, 2006).

The purpose of the present study was to determine whether an orienting response is sufficient to explain the bivalency effect. To do so, we tested whether responding to infrequent *univalent* stimuli produced a similar after-effect as responding to infrequent bivalent stimuli (i.e., the bivalency effect). During three blocks, participants had to perform a parity decision on numerals, a colour decision on symbols, and a case decision on letters. In Blocks 1 and 3 (the pure blocks), all stimuli were univalent. In Block 2 (the mixed block), some letters for the case decisions were slightly modified in order to make them infrequent. We presented two conditions of infrequent stimuli. For the first condition (the bivalent condition), the infrequent stimuli were red or blue letters. As these stimuli had relevant features for two tasks (colour and case decisions), they were bivalent. This condition is a replication of the bivalency effect (Meier et al., 2009; Rey-Mermet & Meier, 2012a; Woodward et al., 2003, 2008). For the second condition (the univalent condition), the infrequent stimuli were green or yellow letters. As these stimuli had relevant features for one task only (i.e., the case decision), they were univalent. However, they varied on the same task dimension as the bivalent stimuli (i.e., colour). Therefore, this condition enables a close comparison with the condition that involves infrequent bivalent stimuli.

We hypothesized that if infrequency is the factor that causes the performance slowing that has been interpreted as the bivalency effect, responding to infrequent univalent stimuli would result in a similar after-effect as the bivalency effect (Notebaert et al., 2009; Notebaert & Verguts, 2011; Nůńez Castellar et al., 2010). In this case, infrequent bivalent and univalent stimuli would slow all subsequent tasks across several trials. Alternatively, if the conflict induced by bivalent stimuli is indeed the critical factor, responding to infrequent univalent stimuli would produce a different pattern than the bivalency effect (Meier et al., 2009; Rey-Mermet & Meier, 2012a, 2012b; Woodward et al., 2003, 2008). Specifically, for the condition with infrequent bivalent stimuli, we expected an enduring performance slowing for all tasks, replicating previous findings (Meier et al., 2009). In contrast, for the condition with infrequent univalent stimuli, we expected a reduced and short-lived slowing.

## Method

### Participants

Participants were 36 volunteers (19 women and 17 men, *M*_age_ = 23.4, *SD* = 3.2) from the University of Bern. Half of them were assigned to the condition with infrequent univalent stimuli and the other half to the condition with infrequent bivalent stimuli. Participants were assigned to each condition alternatingly. The study was approved by the local ethical committee of the University of Bern.

### Materials

For the parity decision, the stimuli were the numerals *1* through *8*, each displayed in black. For the colour decision, the stimuli were the symbols %, #, $, and §, each displayed in either blue or red. For the case decision, the stimuli were the upper- or lowercase consonants *n, p, v, s*, each displayed in black. All stimuli were presented as triplicate (e.g., *777*, &&&, and *nnn*) at the centre of the computer screen in 60-point Times New Roman font (cf. Meier et al., 2009; Woodward et al., 2003). As in our previous bivalency effect studies (Meier et al., 2009, 2013; Rey-Mermet et al., 2013; Rey-Mermet & Meier, 2012a), we created a set of eight infrequent bivalent incompatible stimuli by presenting the letters in either red or blue. We created a set of 16 infrequent univalent stimuli by presenting the letters in either green or yellow (4 letters × 2 cases × 2 colours). From the corresponding set, six infrequent stimuli were determined randomly for each participant.

### Procedure

Participants were tested individually. They were informed that the experiment involved three different tasks: parity decisions about numerals, colour decisions about symbols, and case decisions about letters. They were instructed to respond by pressing one of two computer keys (“b” and “n”) with their left and right index fingers, respectively, for each of the three tasks. The mapping information, printed on paper, was presented below the computer screen throughout the experiment. Participants were informed that, for some of the case decisions, the letters would be modified. In the bivalent condition, they were told that these letters would be presented in either blue or red; in the univalent condition, they were told that these letters would be presented in either green or yellow. All participants were instructed to ignore the modification and to continue making case decisions.

**Figure 1.**
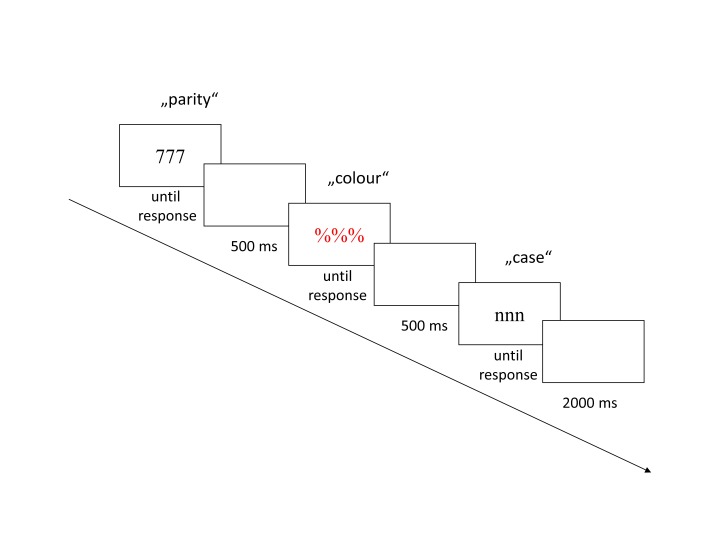
Example of one univalent task triplet. Participants carried out a parity decision (odd vs. even) on numerals, a colour decision (red vs. blue) on symbols, and a case decision (upper- vs. lowercase) on letters. On an infrequent task triplet (not pictured here), the letters were presented in colour. For the bivalent condition, they were printed in either blue or red, for the univalent condition in either green or yellow.

After the instructions, a block of 30 task triplets was presented for practice. Each task triplet required making a parity decision, a colour decision, and a case decision, as illustrated in Figure 1. For each trial, a stimulus was selected randomly and was displayed until the participant responded. Then, the screen blanked for 500 ms before the next stimulus appeared. After each task triplet, an additional blank appeared for 1,500 ms. After the practice block and a brief break, each participant completed three experimental blocks without break between blocks. Block 1 included 32 task triplets, with the first two task triplets serving as “warm-up” sequences which were discarded from the analyses. Blocks 2 and 3 had 30 task triplets each.

For Blocks 1 and 3 (the pure blocks), only frequent univalent stimuli were presented. For Block 2 (the mixed block), frequent univalent stimuli were presented except on 20% of the case decisions in which infrequent stimuli appeared. Trials with infrequent stimuli were evenly interspersed among the 30 task triplets of the block; occurring in every fifth task triplet, specifically in the 3rd, 8th, 13th, 18th, 23rd, and 28th sequences. The entire experiment lasted about 20 min.

### Data analysis

For each participant and each task, the accuracy rates and the median decision times (DTs) for correct responses were computed for each task triplet following an infrequent stimulus in the mixed block and for each corresponding task triplet in the pure Blocks 1 and 3. Specifically, an infrequent stimulus was presented on every fifth task triplet in the mixed block, and this task triplet was designated with the label *N*, with succeeding task triplets labelled *N* + 1, *N* + 2, *N* + 3, and *N* + 4. To account for general training effects, we averaged the data from the pure Blocks 1 and 3 for each task, each task triplet, and each participant. An alpha level of .05 was used for all statistical tests. Greenhouse-Geisser corrections are reported where appropriate and effect sizes are expressed as partial η^2^ values.

## Results

As in the previous bivalency effect studies (Meier et al., 2009; Rey-Mermet & Meier, 2012a, 2012b; Woodward et al., 2003), we first investigated the cost produced by infrequent stimuli. Second, we focused on the main objective of the study, that is, the different after-effects of infrequent bivalent and univalent stimuli.

### Costs of infrequent stimuli

We compared the costs produced by infrequent bivalent and univalent stimuli (i.e., the coloured letters of the case decisions from the task triplets *N* of the mixed block). To do so, we assessed whether performance on infrequent stimuli was worse than performance on the corresponding frequent stimuli (i.e., the black letters of the case decisions from the task triplets *N* + 1 until *N* + 4 of the mixed block), and whether this cost differed between both conditions of infrequent stimuli (bivalent vs. univalent).

#### Decision times

For the bivalent condition, performance was slower on infrequent stimuli (*M* = 1,011 ms, *SE* = 95) than on the corresponding frequent stimuli (*M* = 716 ms, *SE* = 42). Similarly, for the univalent condition, performance was slower on infrequent stimuli (*M* = 1,041 ms, *SE* = 83) than on the corresponding frequent stimuli (*M* = 677 ms, *SE* = 30). A two-way analysis of variance (ANOVA) with Stimulus Frequency (infrequent, corresponding frequent) as a within-subject factor and Condition of Infrequent Stimuli (bivalent, univalent) as a between-subjects factor revealed a significant main effect of stimulus frequency, *F*(1, 34) = 39.81, *p* < .001,η^2^= .54. No other main effect or interaction was significant, *Fs* < 1, *ps* > .52, η^2^ < .01. Thus, performance was significantly slower on infrequent stimuli than on corresponding frequent stimuli, but this cost was similar for both infrequent bivalent and univalent stimuli (295 and 364 ms, respectively).

#### Accuracy

For the bivalent condition, performance was lower on infrequent stimuli (*M* = .89, *SE* = .03) than on the corresponding frequent stimuli (*M* = .95, *SE* = .02). Similarly, for the univalent condition, performance was lower on infrequent stimuli (*M* = .97, *SE* = .02) than on the corresponding frequent stimuli (*M* = .98, *SE* = .005). The two-way ANOVA with Stimulus Frequency (infrequent, corresponding frequent) as a within-subject factor and Condition of Infrequent Stimuli (bivalent, univalent) as a between-subjects factor showed a significant main effect of condition of infrequent stimuli, *F*(1, 34) = 10.65, *p* < .01,η^2^= .24.No other main effect or interaction was significant, *Fs* < 2.75, ps > .11,η^2^ < .07. Thus, accuracy was higher for the univalent condition (*M* = .98, *SE* = .01) than for the bivalent condition (*M* = .92, *SE* = .02). However, no cost was found between the infrequent stimuli and the corresponding frequent stimuli, and this did not differ between both conditions.

Together, these findings show no differences in the costs produced by infrequent bivalent and univalent stimuli. This rules out that a difference in the after-effects resulted from a priori differences in the costs produced by infrequent bivalent and univalent stimuli.

### After-effects of infrequent stimuli

#### Decision times

**Figure 2.**
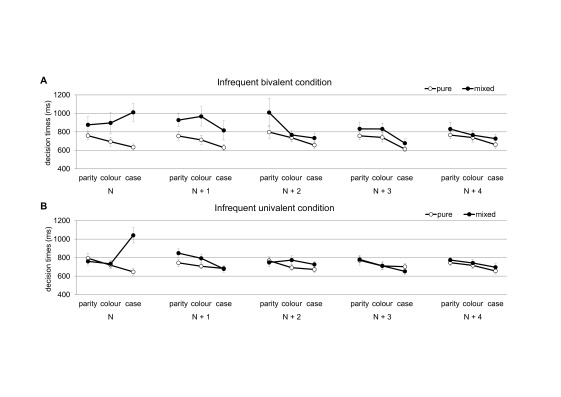
Mean decision times for task triplets from the mixed block (filled circles) and for corresponding task triplets from the pure block (empty circles). Task triplet *N* refers to the triplet containing an infrequent stimulus for the case decision in the mixed block; subsequent task triplets are labelled *N* + 1, *N* + 2, *N* + 3, and *N* + 4, respectively. Error bars represent standard errors. Panel A. Condition with infrequent bivalent stimuli. Panel B. Condition with infrequent univalent stimuli.

The main objective was to examine whether responding to infrequent univalent stimuli would produce a similar after-effect as responding to infrequent bivalent stimuli. To this end, we tested performance on univalent trials following infrequent bivalent stimuli and on those following infrequent univalent stimuli. The most relevant results are the DTs from the univalent trials for each task in the mixed block compared to those from the pure block across the task triplets *N* + 1 until *N* + 4. These results are depicted in Figure 2. We carried out a four-way ANOVA with Block (pure, mixed), Task (parity, colour, case), and Task Triplet (*N* + 1, *N* + 2, *N* + 3, *N* + 4) as within-subject factors and Condition of Infrequent Stimuli (bivalent, univalent) as a between-subjects factor.

The ANOVA showed a significant main effect of task, *F*(1.42, 48.46) = 10.10, *p* < .01,η^2^= .23, and of task triplet, *F*(3, 102) = 4.58, *p* < .01,η^2^= .12. More importantly, there were also a main effect of block, *F*(1, 34) = 23.63, p < .001,η^2^= .41, and a significant interaction between Block and Task Triplet, *F*(3, 102) = 5.57, *p* < .01,η^2^= .14. Thus,performance was slowed after infrequent stimuli and this performance slowing decreased across the task triplets (from 133 ms in *N* + 1 to 42 msin *N* + 4).

Most critically, the two-way interaction between Block and Condition of Infrequent Stimuli was significant, *F*(1, 34) = 7.72, *p* < .01,η^2^= .18. This was caused by a larger performance slowing after infrequent bivalent stimuli, *M* = 110 ms, *SE* = 26, with *t*(17) = 4.19, *p* < .01; than after infrequent univalent stimuli, *M* = 30 ms, *SE* = 12, with *t*(17) = 2.52, *p* < .05. Thus, the bivalency effect (i.e., the slowing after infrequent bivalent stimuli) was significantly larger than the performance slowing after infrequent univalent stimuli.

Moreover, the four-way interaction between Block, Task, Task Triplet, and Condition of Infrequent Stimuli approached significance, *F*(3.85, 130.99) = 2.05, *p* < .09,η^2^= .06, suggesting that the performances lowing after infrequent bivalent and univalent stimuli persisted differently across tasks and task triplets. Due to the theoretical and practical interest, we followed up this interaction by conducting three-way repeated-measures ANOVAs for each Condition of Infrequent Stimuli (bivalent and univalent), with the factors Block (pure, mixed), Task (parity, colour, case), and Task Triplet (*N* + 1, *N* + 2, *N* + 3, *N* + 4).

For the bivalent condition, the three-way ANOVA revealed a significant main effect of block, *F*(1, 17) = 17.59, *p* < .01,η^2^= .51, of task, *F*(1.37, 22.32) = 5.88, *p* < .05,η^2^= .26, and of task triplet, *F*(3, 51) = 3.07, *p* < .05,η^2^= .15, as well as a significant interaction between Block and Task Triplet, *F*(3, 51) = 3.75, *p* < .01,η^2^= .18. No other interaction was significant, *Fs* < 1.84, *ps* > .16,η^2^< .10. Thus, the bivalency effect decreased across the task triplets from 204 ms to 106 ms, to 77 ms, and to 52 ms for *N* + 1, *N* + 2, *N* + 3, and *N* + 4, respectively (see Panel A of Figure 2). In follow-up two-way repeated-measures ANOVAs with the factors Block (pure, mixed) and Task (parity, colour, case), the main effect of block was significant for the task triplets *N* + 1 until *N* + 3; *N* + 1: *F*(1, 17) = 18.70, *p* < .001,η^2^= .52; *N* + 2: *F*(1, 17) = 4.94, *p* < .05,2 = .22; *N* + 3: *F*(1, 17) = 5.86, p < .05, 2 = .26; and marginally significant for the task triplet *N* + 4: *F*(1, 17) = 3.55, *p* = .08,η^2^= .17. Across the four task triplets, no interaction between Block and Task was significant, *Fs* < 1.34, *ps* > .27,η^2^< .07. Thus, the bivalency effect decreased across task triplets, irrespective of the tasks, but remained significant up to the task triplets *N* + 4. This finding replicates the persistence of the bivalency effect (Meier et al., 2009).

For the univalent condition, the three-way ANOVA with the factors Block (pure, mixed), Task (parity, colour, case), and Task Triplet (*N* + 1, *N* + 2, *N* + 3, *N* + 4) showed a significant main effect of block, *F*(1, 17) = 6.35, p < .05,η^2^= .27, and of task, *F*(1.51, 25.62) = 4.35, p < .05,η^2^= .20. More importantly, there were also significant interactions between Block and Task Triplet as well as between Block, Task, and Task Triplet, *F*(3, 51) = 3.27, *p* < .05,η^2^= .16, and *F*(6, 102) = 2.65,p < .05,η^2^= .13, respectively. No other main effect or interaction was significant, *Fs* < 1.88, *ps* > .14, 2 < .10. Thus, the performance slowing following green or yellow letters decreased rapidly both across tasks and task triplets (see Panel B of Figure 2). In follow-up two-way repeated-measures ANOVAs with Block (pure, mixed) and Task (parity, colour, case), the main effect of block was significant for the task triplets *N* + 1 and *N* + 2; *N* + 1: *F*(1, 17) = 12.81, *p* < .01,η^2^= .43; and *N* + 2: *F*(1, 17) = 7.07, *p* < .05,η^2^= .29, but not for subsequent task triplets, *Fs* < 2.03, *ps* > .17,η^2^< .11. Thus, the performance slowing following infrequent univalent stimuli decreased across the first two task triplets (62 and 40 ms, respectively) and was no longer significant at triplets *N* + 3 and *N* + 4 (-13 and 32 ms, respectively). Moreover, at both task triplets *N* + 1 and *N* + 2, an interaction between Block and Task was observed, *F*(1.51, 25.72) = 3.08, *p* < .08,η^2^= .15, and *F*(2, 34)= 4.39, p < .05,η^2^= .20, respectively. For the task triplets *N* + 1, performance was significantly slowed on parity and colour decisions, but not on case decisions; parity: *M* = 105 ms, *SE* = 42, with *t*(17) = 2.49, *p* < .05; and colour: *M* = 86 ms, *SE* = 27, with *t*(17) = 3.18, p < .01; but case: *M* = -6 ms, *SE* = 26, with *t*(17) = -0.23, *p* = .82. For the task triplets *N* + 2, performance was significantly slowed on colour and case decisions, but not on parity decisions; colour: *M* = 82 ms, *SE* = 27, with *t*(17) = 3.02, *p* < .01; and case: *M* = 55 ms, *SE* = 19, with *t*(17) = 2.84, *p* < .05; but parity: *M* = -19 ms, *SE* = 28, with *t*(17) = -0.67, *p* = .51. This indicates that infrequent univalent stimuli produce a shorter-lived and more task-specific effect than infrequent bivalent stimuli.

#### Accuracy

**Figure 3.**
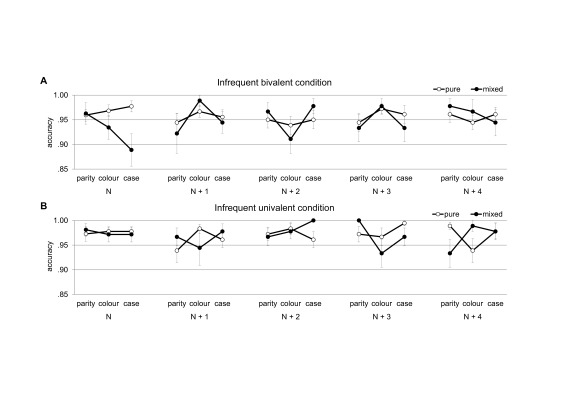
Mean accuracy rates for task triplets from the mixed block (filled circles) and for corresponding task triplets from the pure block (empty circles). Task triplet *N* refers to the triplet containing an infrequent stimulus for the case decision in the mixed block; subsequent task triplets are labelled *N* + 1, *N* + 2, *N* + 3, and *N* + 4, respectively. Error bars represent standard errors. Panel A. Condition with infrequent bivalent stimuli. Panel B. Condition with infrequent univalent stimuli.

The accuracy rates are depicted in Figure 3. Despite the fact that they were close to ceiling, we also conducted a four-way ANOVA with Block (pure, mixed), Task (parity, colour, case), and Task Triplet (*N* + 1,N + 2, *N* + 3, *N* + 4) as within-subject factors and Condition of Infrequent Stimuli (bivalent, univalent) as a between-subjects factor. This ANOVA revealed a significant three-way interaction between Task, Task Triplet, and Condition of Infrequent Stimuli, *F*(4.50, 152.98) = 2.41, *p* < .05,η^2^= .07.^1^ No other main effect or interaction was significant, *Fs* < 2.14, *ps* > .14,η^2^< .06. Thus, there was no main effect or interaction involving block, which indicates that no speed-accuracy trade-off compromised the critical DTs effects.

## Discussion

The purpose of the present study was to test whether the bivalency effect results from an orienting response towards infrequent bivalent stimuli. To this end, we compared the after-effect of responding to infrequent bivalent stimuli (i.e., the bivalency effect) to the after-effect of responding to infrequent but univalent stimuli. For the infrequent bivalent stimuli condition, the results showed a performance slowing for all four task triplets, and this effect lasted at least for 20 s (required for making four task triplets, i.e., 12 decisions, each requiring approximately 750 ms, plus eight blanks of 500 ms, plus four blanks of 2,000 ms). This indicates a long-lasting bivalency effect, replicating previous findings (cf. Meier et al., 2009; Rey-Mermet et al., 2013). In contrast, for the infrequent univalent stimuli condition, the results showed that performance was slowed for the first two tasks immedia-tely following infrequent univalent stimuli. For the subsequent task triplet, performance was still slowed on the tasks sharing features with the infrequent univalent stimuli. However, compared to the infrequent bivalent condition, no longer-lasting effect materialized.

It must be noted that although different after-effects were found after infrequent bivalent and univalent stimuli, there was no difference in the costs produced by infrequent bivalent and univalent stimuli. Thus, responding to infrequent bivalent and univalent stimuli did not result in differences within the trial itself, but it did result in differences for subsequent trials. This rules out the possibility that different after-effects resulted from a priori differences between infrequent bivalent and univalent stimuli. This is important because infrequent bivalent stimuli had task-relevant features for another task (i.e., the features “red” and “blue” for the colour decision), and thus they could have attracted more attention than infrequent univalent stimuli. Consequently, they could have produced a larger orienting response for the trial in which they occur as well as for the subsequent trials. In this case, the larger and long-lasting bivalency effect would have simply resulted from a larger orienting response. However, the present results showed no differences in the costs produced by infrequent bivalent and univalent stimuli. This suggests that the orienting response is similar for infrequent bivalent and univalent stimuli.

More importantly, the findings of the present study showed that the infrequence of univalent stimuli results in an orienting response that was sufficient to slow down the two subsequent decisions (i.e., the parity and colour decisions of task triplets N + 1). These results indicate that the bivalency effect is more than an orienting response. Moreover, they extend previous findings that the infrequency of an event has an impact on subsequent performance (cf. Barcelo et al., 2006; Notebaert et al., 2009; Notebaert & Verguts, 2011; Nůńez Castellar et al., 2010). In those previous studies, a performance slowing was found on the trial following an infrequent event, irrespective of whether this infrequent event was an oddball tone, a correct response, or an error (cf. Barcelo et al., 2006; Notebaert et al., 2009). Here, we also found that performance was slowed after an infrequent event, even when the event was an infrequent univalent stimulus. Thus, investigating the after-effects of a large variety of infrequent events, such as errors, infrequent correct responses, oddball tones, or infrequent univalent stimuli is a promising avenue for future research in order to clarify the contribution of the orienting response to performance.

It is also noteworthy that in the present study, the infrequence of univalent stimuli also slowed down some subsequent decisions (i.e., the colour and case decisions of task triplet *N* + 2), but only on those tasks that had overlapping features with the infrequent univalent stimuli. Thus, it is possible that because of this overlap, representations of infrequent stimulus features were activated, and this interfered with current processing, slowing down performance (see Allport & Wylie, 1999, 2000). However, this pattern of slowing was not predicted and its interpretation is post-hoc and somewhat speculative. Further research is necessary to replicate this specific pattern and to provide a more solid foundation for this interpretation.

Together, the present results demonstrate that the short-lived and - at least partly - task-specific after-effect triggered by infrequent univalent stimuli is different from the robust and long-lasting bivalency effect. Therefore, the infrequence of bivalent stimuli and its resulting orienting response may explain the performance slowing on the first trials following bivalent stimuli, but not the whole bivalency effect across the four task triplets. Consequently, responding to infrequent bivalent stimuli results in an additional process that is related to the conflict induced by bivalent stimuli. One possible additional process may be the reactivation of the more demanding context created by bivalent stimuli, such as proposed in the episodic context binding account (Meier et al., 2009, 2013; Rey-Mermet & Meier, 2012a, 2012b; cf. also Meier & Rey-Mermet, 2012a).

More generally, the study of the bivalency effect extends cognitive control research in which conflict is induced by incongruent stimuli (i.e., stimuli with relevant features for two different responses, such as in the Stroop and Flanker tasks^2^; cf. Botvinick et al., 2001; Egner, 2007). Typically, performance is slowed on incongruent stimuli compared to congruent stimuli (i.e., stimuli with relevant features for one response). This congruence effect is smaller after incongruent stimuli than after congruent stimuli. This reduction in congruence effect has been labelled *congruence sequence effect* (e.g., Botvinick et al., 2001; Clayson & Larson, 2011; Egner, 2007; Schlaghecken & Martini, 2012). The congruence effect is also smaller when the proportion of incongruent stimuli increases in the block. This second reduction has been labelled *proportion congruence effect* (e.g., Gratton, Coles, & Donchin, 1992; Hommel, 1994; Logan & Zbrodoff, 1979; Lowe & Mitterer, 1982). Previous research has shown that the congruence sequence effect may be dependent on stimulus and response overlap, affecting only those subsequent trials that shared stimulus or response features with incongruent stimuli (Akçay & Hazeltine, 2008; Hazeltine, Lightman, Schwarb, & Schumacher, 2011; Mayr, Awh, & Laurey, 2003; but see Ullsperger, Bylsma, & Botvinick, 2005). The congruence sequence effect may also be specific to the source of conflict, only affecting the subsequent trials that shared features with the conflict induced by incongruent stimuli. That is, responding to incongruent Stroop stimuli induced a congruence sequence effect when the subsequent stimuli were Stroop stimuli, but not when they were Flanker stimuli (e.g., Egner, 2008; Egner, Delano, & Hirsch, 2007; Funes, Lupiáńez, & Humphreys, 2010; Notebaert & Verguts, 2008; Schlaghecken, Refaat, & Maylor, 2011; but see Freitas, Bahar, Yang, & Banai, 2007; Kunde & Wühr, 2006). For the proportion congruence effect, previous research has shown that it may be affected by the proportion of incongruent trials at the item level. In this research, participants were usually asked to perform a Stroop task with at least four colours split in two binary pairs. For one binary pair (e.g., red and blue), the trials were mostly incongruent, whereas for the other binary pair (e.g., green and yellow), the trials were mostly congruent. Each colour word was printed in its own colour for congruent items and in the colour of the other member of its pair for incongruent items. Those items that were “mostly incongruent” showed a smaller congruence effect than those items that were “mostly congruent” (Blais, Robidoux, Risko, & Besner, 2007; Jacoby, Lindsay, & Hessels, 2003; but see Bugg, McDaniel, Scullin, & Braver, 2011, for list-wide proportion congruence effects). These findings lead to the conclusion that both the congruence sequence and the proportion congruence effects are - to some extent - the result of binding processes operatingacross stimulus-, response-, and/or task-representations (e.g., Blais et al., 2007; Hommel, 2004; Mayr et al., 2003; Verguts & Notebaert, 2009). In contrast, the bivalency effect is not affected by the overlap of stimulus-, response-, and task-representations. In fact, it occurs on univalent trials, which have no stimulus-, response- and task-feature overlap with the previously encountered bivalent stimuli (Rey-Mermet & Meier, 2012a; Woodward et al., 2003). Thus, the bivalency effect goes beyond stimulus, response, and task representations, but rather includes the context representation (see Meier & Rey-Mermet, 2012a). Therefore, investigating the bivalency effect reflects a new way to explore cognitive control.

In a recent framework, Braver and colleagues have differentiated between proactive and retroactive cognitive control (Braver, 2012; Braver, Gray, & Burgess, 2007). Proactive control reflects the sustained and anticipatory maintenance of task-relevant representations, and is initiated before a conflict is encountered. In contrast, reactive control reflects the transient stimulus-driven reactivation of task representations after a conflict was encountered. The bivalency effect clearly contains a reactive component because it reflects an adjustment of cognitive control following the conflict induced by bivalent stimuli. However, due to the long-lasting nature of the bivalency effect across trials, it may also reflect a proactive control process in anticipation of the occurrence of the next bivalent stimulus. We tested this possibility in a recent study with a similar set-up as the present study, but in order to induce proactive control, we asked participants to deliberately search for (infrequent) bivalent stimuli (Meier & Rey-Mermet, 2012b). Moreover, they were instructed to respond with a different key-press (i.e., the “h” key) whenever they noticed such an (infrequent) bivalent stimulus. The results showed a performance slowing for the first task triplet that immediately followed the bivalent stimuli, reflecting an orienting response (cf. Notebaert et al., 2009; Notebaert & Verguts, 2011; Nůńez Castellar et al., 2010). In addition, on subsequent task triplets, a performance slowing was found but only for those univalent stimuli which shared relevant features with bivalent stimuli (i.e., the colour and case decisions). Therefore, inducing proactive control does not result in the same pattern of slowing as the bivalency effect, which suggests that the bivalency effect is mainly driven by reactive control.

To summarize, the findings of the present study show that the adjustment of cognitive control underlying the bivalency effect results from the conflict induced by bivalent stimuli and not simply from the occurrence of infrequent stimuli. For the example of encountering a traffic light with an infrequent pattern, it suggests that for a blinking red light (infrequent univalent condition), you will probably increase control at this particular junction and at the subsequent junction with a “normal” traffic light. In contrast, when you encounter a traffic light with red and green lights lit at once (infrequent bivalent condition), you will probably show a longer lasting increase of cognitive control. According to the episodic context binding account, this is because in this situation, encountering univalent stimuli will reactivate the previous conflict-loaded context.

## Footnotes

1 To further investigate the significant interaction between Task, Task Triplet, and Condition, we averaged the data across the two block types and we carried out follow-up two-way repeated-measures ANOVAs for each Condition of Infrequent Stimuli, with the factors Task (parity, colour, case) and Task Triplet (*N* + 1, *N* + 2, *N* + 3, *N* + 4).For the univalent condition, the ANOVA revealed no significant main effect or interaction, *Fs* < 1.12, *ps* > .35,η^2^< .06. In contrast, for the bivalent condition, the two-way interaction between Task and Task Triplet approached significance, *F*(6, 102) = 2.05, *p* < .07,η^2^= .11. Therefore, we conducted a follow-up one-way repeated-measures ANOVA with the factor Task Triplet (*N* + 1, *N* + 2, *N* + 3, *N* + 4) for each task separately. Only for the colour decisions, the one-way ANOVA showed a significant main effect of task triplet, *F*(3, 51) = 3.52, *p* < .05,η^2^= .17,with a significant cubic component, *F*(1, 17) = 6.01, *p* < .05,η^2^= .26. Thus, for the bivalent condition accuracy on colour decisions was higher in the task triplets *N* + 1 and *N* + 3 (both *Ms* = .98, *SE* = .01) than in the task triplets *N* + 2 and *N* + 4 (*M* = .93, *SE* = .02, and *M* = .96,*SE* = .02, respectively).

2 In the Stroop task, stimuli are colour words that are printed in colour (e.g., the word *red* printed in red or blue), and participants are asked to indicate the colour of the word. Incongruent Stroop stimuli are colour words that are printed in a different colour (e.g., the word *red* printed in blue). In the Flanker task, stimuli consist of strings of letters (e.g., *HHH* or *SHS*), and participants are asked to indicate the identity of the central letter. Incongruent Flanker stimuli are letter strings in which the central letter is different from the flanking letters (e.g., *SHS*).
